# Occurrence and analysis of microplastics in municipal wastewater, Poland

**DOI:** 10.1007/s11356-024-34488-z

**Published:** 2024-07-30

**Authors:** Paulina Ormaniec

**Affiliations:** Department of Environmental Technologies, Faculty of Environmental Engineering and Energy, Cracow University of Technology, Warszawska 24, Cracow, Poland

**Keywords:** Microplastics, Municipal wastewater, Micropollutants, Wastewater treatment plant

## Abstract

Microplastics are a growing environmental threat and wastewater treatment plants have been identified as significant conduits for these pollutants. This study addresses microplastic loading in the influent of a large urban wastewater treatment plant, presenting a detailed analysis of their prevalence and characteristics. Our findings reveal a concentration of 4.09 microplastic particles per litre in the tributary. We performed a detailed statistical comparison of the microplastic particles, categorising them by shape, size, colour, and polymer type. Using Fourier transform total reflectance infrared spectroscopy, we identified 13 different polymer types, with polyethylene terephthalate, rubber, and polyethylene predominating. The analysis showed that textile fibres, mainly from clothing, are the most prevalent form of microplastic in wastewater, followed by fragments from the breakdown of larger plastic objects and films. This research highlights the critical need for strategic interventions to mitigate microplastic pollution at municipal sources.

## Introduction

Microplastics (MPs) are commonly defined as plastic particles smaller than 5 mm in diameter. This definition, highlighted by Thompson, forms the basis for scientific research and environmental monitoring (Thompson 2015). Verschoor in his report highlights the need for precise size limits for MPs, which is key to understanding their impact on the environment and human health (Verschoor 2015). Kooi et al. investigated the multidimensionality of MPs in different environments, highlighting the diversity of polymer types and sizes depending on origin and application (Kooi et al. 2021). These diverse perspectives highlight the complexity of the MPs problem and the need for further research into their environmental and health impacts. MPs are present in a wide variety of habitats, posing a global environmental problem. According to a study by Cole et al., these particles can be found in marine ecosystems around the world, indicating their persistence and capacity for long-term transport (Cole et al. 2011). He et al. showed that MPs are also present in rivers, suggesting that inland waters are a significant route for their migration (He et al. 2020). MPs have also been found to be present in the atmosphere and soil, which expands the understanding of their impact on a variety of natural environments, potential risks to human health and biota, and the transport of these particles in the environment (Chen et al. 2020; Guo et al. 2020). The impact of MPs on living organisms depends on the physical and chemical nature of the particles. The chemical properties depend on the composition of the plastic. In addition to the basic MPs polymer, they have additives and fillers, such as plasticizers, antioxidants, lubricants, or dyes. Among the physical properties of MPs, it is important to note the ratio of MPs’ specific surface area to their volume. Due to their high specific surface area and strong hydrophobicity, MPs become capacious adsorbents of pollutants and microorganisms (Zoltovana et al. 2022).

Wastewater treatment plants (WWTPs) are considered one of the main sources of MPs in the environment. Studies have been conducted in many countries to identify WWTPs as a source of MPs in the environment. The lack of standardisation of sampling and analysis of isolated MPs greatly affects the range of MPs identified. In addition, analytical limitations have a huge impact on the reported MPs concentrations in the samples. For example, MPs identification using ATR-FTIR spectroscopy allows for the identification of material larger than 80 µm. However, using micro-Raman spectroscopy increases the detection range to 5 µm (Liu et al. 2021). On the basis of review papers, it has been found that the content of MPs in raw wastewater ranges from a few to several thousand particles per litre (Liu et al. 2021; Mesquita et al. 2023; Nafea et al. 2023). For example, a municipal WWTP located in northern Italy contains in its raw wastewater 2.5 ± 0.3 MP/L. The daily flow rate at this treatment plant is 4.0 × 10^5^ (with a served population: 1,200,000) (Magni et al. 2019). In contrast, raw wastewater collected from one Danish WWTP, which manages mainly domestic wastewater, contained 18,285 MP/L. At the other WWTPs in this study, the MP content ranged from 2223 to 10,044 MPs per litre of wastewater. All WWTPs perform biological N and P removal based on activated sludge technology (Simon et al. 2018). In the case of treated wastewater, we encounter similar whanias of MPs content in the tested wastewater. MPs particles were sampled at different stages of the treatment process at a large WWTP on the River Clyde in Glasgow. The study presented shows that the treated wastewater contained 0.25 (± 0.04) MP/L. The plant serves a population of approximately 650,000 and produces an average of 260,954 m^3^ of treated effluent per day which is discharged into Glasgow’s main waterway, the River Clyde (Murphy et al. 2016). However, the researchers also present much higher concentrations of MPs in treated wastewater. For example, a municipal WWTP located near Madrid (Spain) based on biological wastewater treatment discharges an average of 10.7 ± 5.2 MP/L. The wastewater is discharged into the Henares River in the Tagus basin (Edo et al. 2020). In municipal WWTPs, the content of MPs is also tested in sewage sludge. The content of MPs in sewage sludge is closely related to the characteristics of MPs in raw wastewater. Studies carried out at municipal WWTPs in the UK show that, depending on the WWTPs studied, the MP content in sewage sludge ranges from 301 to 10,380 MP particles per kilogram of sludge dry weight (Horton et al. 2021).

It has been shown that despite the high efficiency of WWTPs, MPs are still found in wastewater. It is estimated that municipal WWTPs remove about 40 to 99.99% of MPs. This discrepancy is due to differences in the wastewater treatment technology used. It is calculated that 63 to 98% of MPs are removed during primary treatment processes, and 7 to 30% during secondary treatment (Turan et al. 2021). The removal of MPs largely depends on their size and hydrophobicity. The removal efficiency of MPs largely depends on the type and biodegradability of the material from which they are made, too. Among wastewater treatment mechanisms, technologies that promote the removal of MPs stand out. For example, studies indicate that filter-based treatment processes are satisfactorily effective in removing MPs from wastewater. Larger MPs are easily and effectively captured during primary sedimentation processes. Data from Dutch WWTPs indicate a 90% removal efficiency of MPs by activated sludge. However, it should not be claimed that biochemical decomposition of MPs will occur during biological treatment. Some of them may be covered by the biological membrane or undergo a change in density, causing them to sink to the bottom (Liu et al. 2021; Singh et al. 2021).

This paper focuses on MPs contamination in a municipal WWTP in southern Poland. The abundance, colour, shape, size, and type of MPs occurring in the influent from WWTP were examined. In addition, on the basis of the results obtained, an attempt was made to identify potential sources of MPs flowing into the municipal WWTP. Despite the relatively large number of studies that have investigated the content of MPs in municipal WWTPs, it is of great importance to gain an understanding of the sources of these particles in the wastewater that is being treated.

## Study materials and methods

### Study materials

The target WWTP is located in a typical densely populated city in southern Poland. The treatment plant consists of a mechanical part, a biological part, and a sludge treatment line with a biogas installation. During the dry season, the average daily volume of wastewater is 165,000 m^3^‧day^−1^. The treatment plant was designed for a population equivalent to 680,000.

A pump-and-filter system was used to collect wastewater samples, which guaranteed continuous sampling. The pumped wastewater (100 L) was directed directly to steel screens with appropriate mesh sizes (5000 µm, 1000 µm, 500 µm, 160 µm, 45 µm). The material collected from the sieves was transported to the laboratory for further analysis.

### Study methods

The sample processing procedure was based on previously described methods for isolating MPs from wastewater (Lares et al. 2018; Lv et al. 2019; Quinn et al. 2016). The fraction was dried at 90 °C for 24 h. Organic matter was removed using Fenton’s reagent. A mixture of the test sample, 30% hydrogen peroxide (H_2_O_2_) and 0.05 M ferrous sulphate solution (FeSO_4_), was heated to 75 °C and stirred for 30 min (pH = 4.5). After the 30-min reaction, a new portion of hydrogen peroxide (20 cm^3^) was added and the solution was heated and stirred again for 30 min. The process was repeated until the organic matter visible to the naked eye was removed. The inorganic matter was removed by density separation using an aqueous zinc chloride (ZnCl_2_) solution with a density of approximately 1.7 g/cm^3^. The test sample was mixed with the saturated solution and left for 24 h. The top layer with suspended particles was filtered through a cellulose filter. The residue on the filter was washed several times with distilled water. The isolated material was dried at 90 °C and further identified.

The dried particles were first subjected to physical analysis. Particle morphology was examined using a stereomicroscope (Leica EZ4 W × 35). Samples were analysed by Fourier transform infrared spectroscopy (FTIR) using an ATR (attenuated total reflectance) attachment in the range 450–4000 cm^−1^, and 64 scans were averaged for each spectrum at room temperature (FTIR, PerkinElmer Spectrum Two). Recording the IR absorption spectra of MP and identifying their specific functional groups confirms the type of MP. The identification of specific functional groups was based on the use of the AssureID^TM^ analysis software, which is the most comprehensive solution that allowed the infrared spectra of the samples to be generated and compared with reference spectra.

## Result and discussion

### Characteristics of microplastics

The characterisation of the isolated MPs was based on two steps: physical and chemical characterisation. The objective of the physical characterisation was to identify the particles in terms of size, shape, and colour of the MPs. Chemical identification was based on the determination of the polymer from which the plastic was created. Analysis of the data obtained provides a better understanding of potential sources of MPs in a municipal WWTP.

**Fig. 1:**
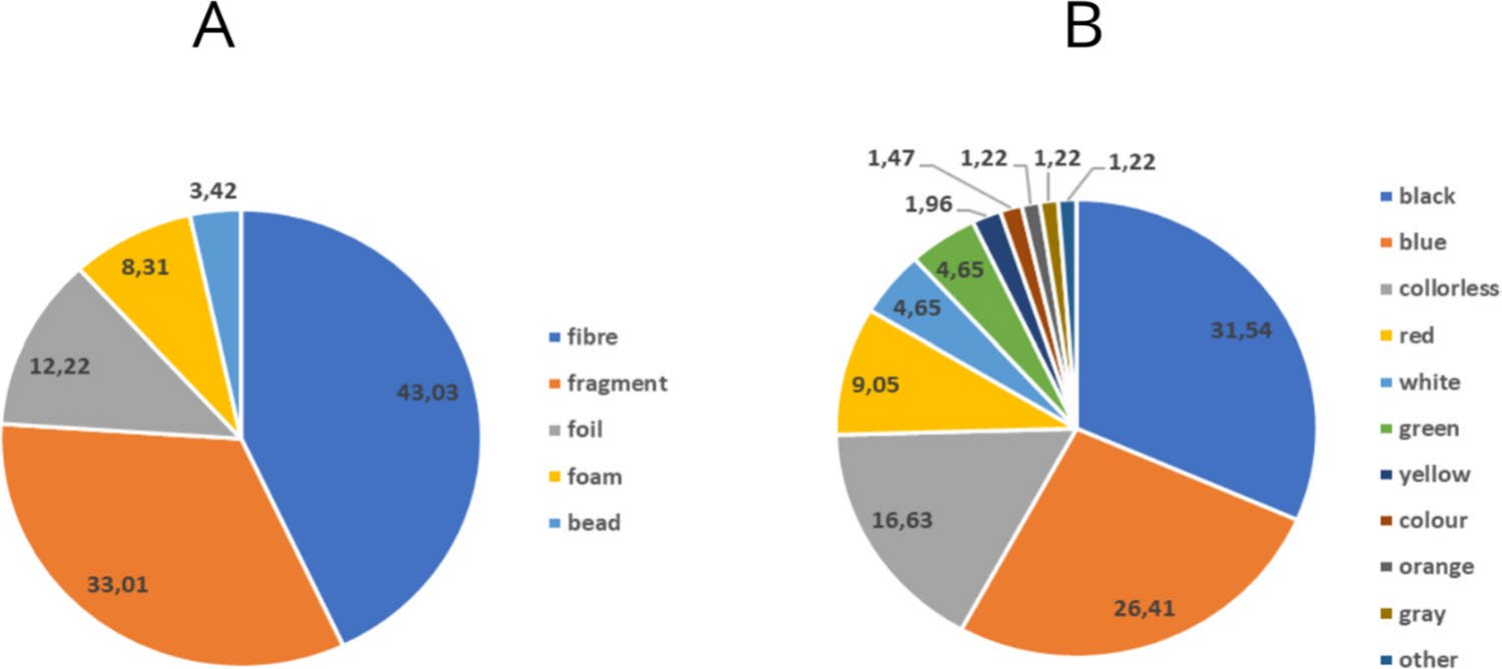
Average percentages of shapes (**A**) and colours (**B**) of MP particles [%]

The number of MPs identified in the raw wastewater was 4.09 MPs/L. In comparison, one other European (Italy) WWTP receives a daily load of MPs of 2.5 ± 0.3 MPs/L (Murphy et al. 2016). Physical analysis of the isolated particles allowed MPs to be classified into five basic groups according to their shape: fragment, film, fibre, foam, and bead. More than 43% of all isolated particles were identified as fibres. The overwhelming prevalence of synthetic fibres has also been confirmed in other municipal WWTPs, which directly demonstrates that perhaps fibres derived from synthetic fabrics may be the largest source of MPs in raw wastewater (Lares et al. 2018; Van Do et al. 2022). Just over 33% were classified as fragments, most likely from the break-up of larger pieces of plastic (e.g., containers, bottles). This was followed by the identification of 12.22% pieces of film, 8.31% pieces of foam, and 3.42% beads. The colour analysis identified 13 different colours. The most common colour was black (31.54%), followed by blue (26.41%), colourless (16.63%), and red (9.05%) (Fig. 1). Chemical analysis, based on the identification of the FTIR spectrum of each piece, showed that 44.01% of the pieces were polyethylene terephthalate (PET), which directly coincides with the dominance of fibres in shape identification. This was followed by the identification of 19.07% rubber pieces, 14.15% polyethylene (PE) pieces, 8.56% polypropylene (PP) pieces, 2.93% polystyrene (PS) pieces, 2.69% polyurethane (PUR) pieces, and 2.93% silicone pieces (including poly(dimethylsiloxane) (PDMS)). The remaining 5.62% were identified as polycyclohexylenedimethylene terephthalate (PCT), polyamide (nylon), polyethylene/polypropylene copolymer (PE/PP), epoxy resin (EP), polyvinyl chloride (PVC), polyacrylamide (PAM), polyacrylonitrile (PAN), and polyethyl acrylate.

**Fig. 2:**
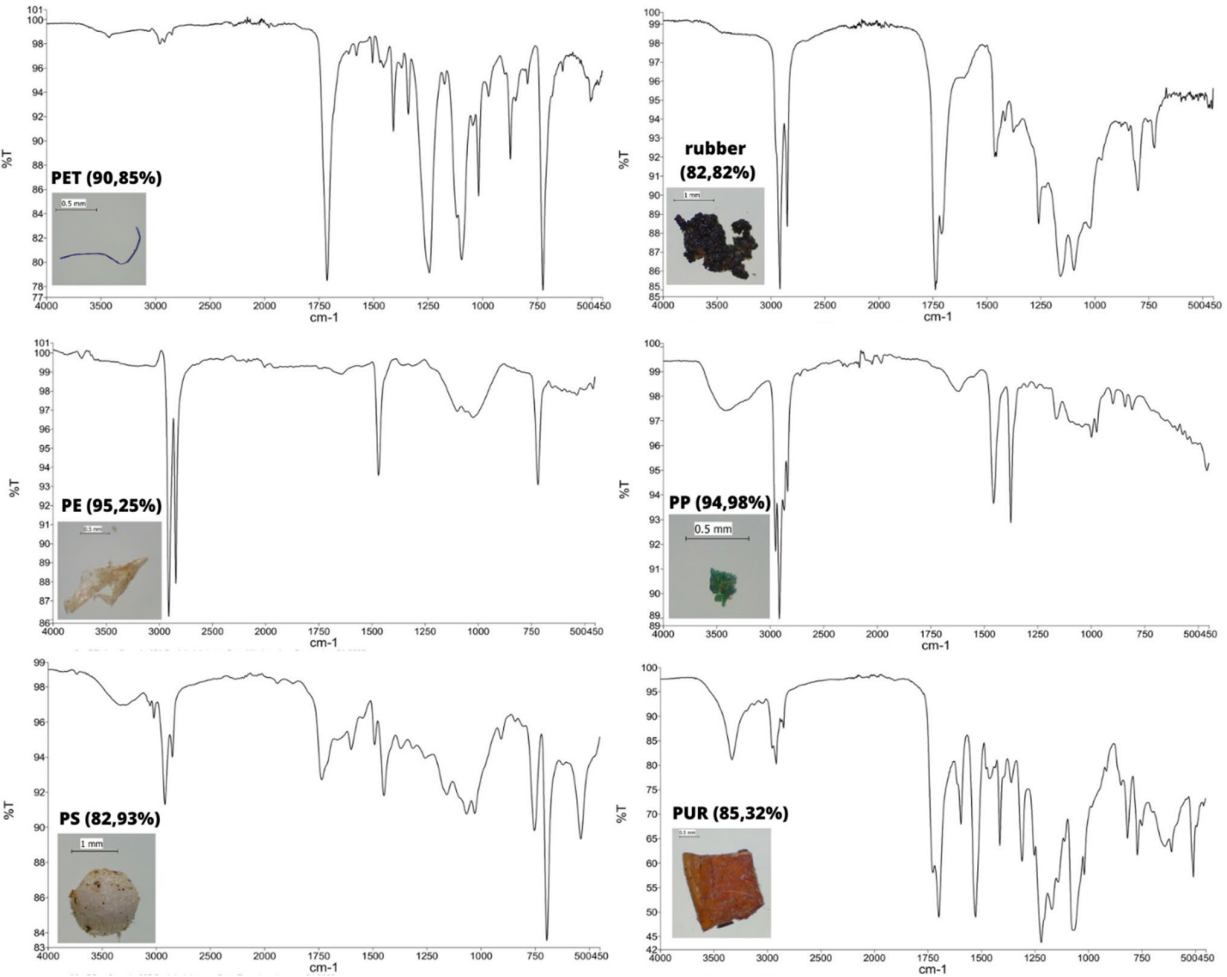
ATR-FTIR spectra identified for PET, rubber, PE, PP, PS, and PUR

Figure 2 shows the ATR-FTIR spectra of the six most common polymers found in wastewater. Identification was performed using a reference library. Next to the name/symbol of the identified polymer, the percentage overlap of the obtained spectrum with the reference spectrum of the presented substance is shown. In addition, images of the isolated MPs for which the spectrum was obtained, are shown next to the spectrum. The images show PET fibre, rubber foam, PE film, PP foam, PS bead, and a PUR fragment (Fig. 2).

**Fig. 3:**
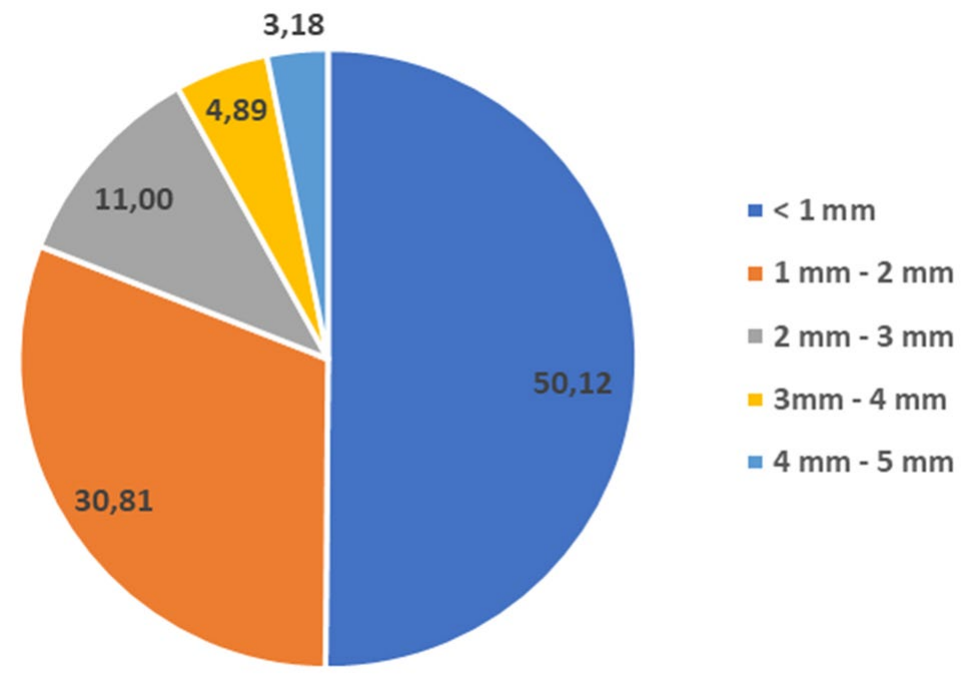
Size distribution of MPs in wastewater [%]

For the size analysis, a granulometric classification method was used, where each MPs particle was assigned to its corresponding size category with a division every 1 mm. This method of segmentation allowed a detailed analysis of the particle size distribution in the samples, enabling a precise study of their heterogeneity. This method ensured systematic and reproducible measurements, crucial for the objective interpretation of the research results. Based on the size categorisation of MPs, according to International Standards, almost 50% of all MPs fall into the category of ‘large microplastics’ (sizes between 1 and 5 mm). This shows directly that MPs in the entire size range enter the municipal WWTP along with the raw wastewater (International Organization for Standarization 2023). Figure 3 shows the percentage of MPs according to the breakdown adopted earlier.

### Characterisation of potential sources of microplastics

The characterisation of the sources of MPs in the environment is a key element in understanding their cycling and impact on ecosystems. The diversity of sources of these pollutants, including consumer products, industrial waste, and municipal runoff, significantly affects the content and composition of MPs in raw wastewater. This variability is strongly linked to the location of WWTPs, which receive waste from different urban and industrial areas. WWTPs situated in regions with a high concentration of industrial activity exhibited elevated concentrations of MPs in their raw wastewater, in comparison to those situated in less-developed urban areas (Conley et al. 2019). This evidence underscores the significance of localised analysis of MP sources, which can facilitate a more comprehensive understanding and more effective management of these pollutants.

MPs are typically classified into two main categories: primary and secondary. Primary MPs are those produced as small molecules for specific applications, such as in cosmetics, industry, or medicine. In contrast, secondary MPs are formed by the decomposition of larger plastic components that degrade when exposed to environmental factors, such as ultraviolet radiation, friction, or water. This degradation process results in the gradual fragmentation of larger plastic particles into smaller and smaller fragments, which are collectively referred to as MPs (Song et al. 2024). Both primary and secondary MPs present a significant environmental threat due to their long-lasting nature and their capacity to accumulate in food chains. The shape of the MPs was used to subdivide the isolated MPs. On the basis of the literature, it was posited that primary MPs would include beads. The granules are frequently utilised as a raw material in the manufacture of plastic products. They are produced in a specific manner, with the intention of creating small, uniform shapes, which are subsequently processed into a variety of objects. Furthermore, these granules can be utilised directly in cosmetic products as abrasive particles (Dąbrowska et al. 2022). While secondary MPs were characterised as fragments, films, fibres, and foams. Given their structure, namely unregulated and jagged structures, these particles were postulated to originate from the breakdown of larger plastics. The aforementioned breakdown indicates that in excess of 3% of all MPs isolated were classified as MPs deliberately produced in microscopic sizes. This implies that in excess of 90% of the influent to the WWTP is secondary MPs, originating from the breakdown of larger plastics.

**Fig. 4:**
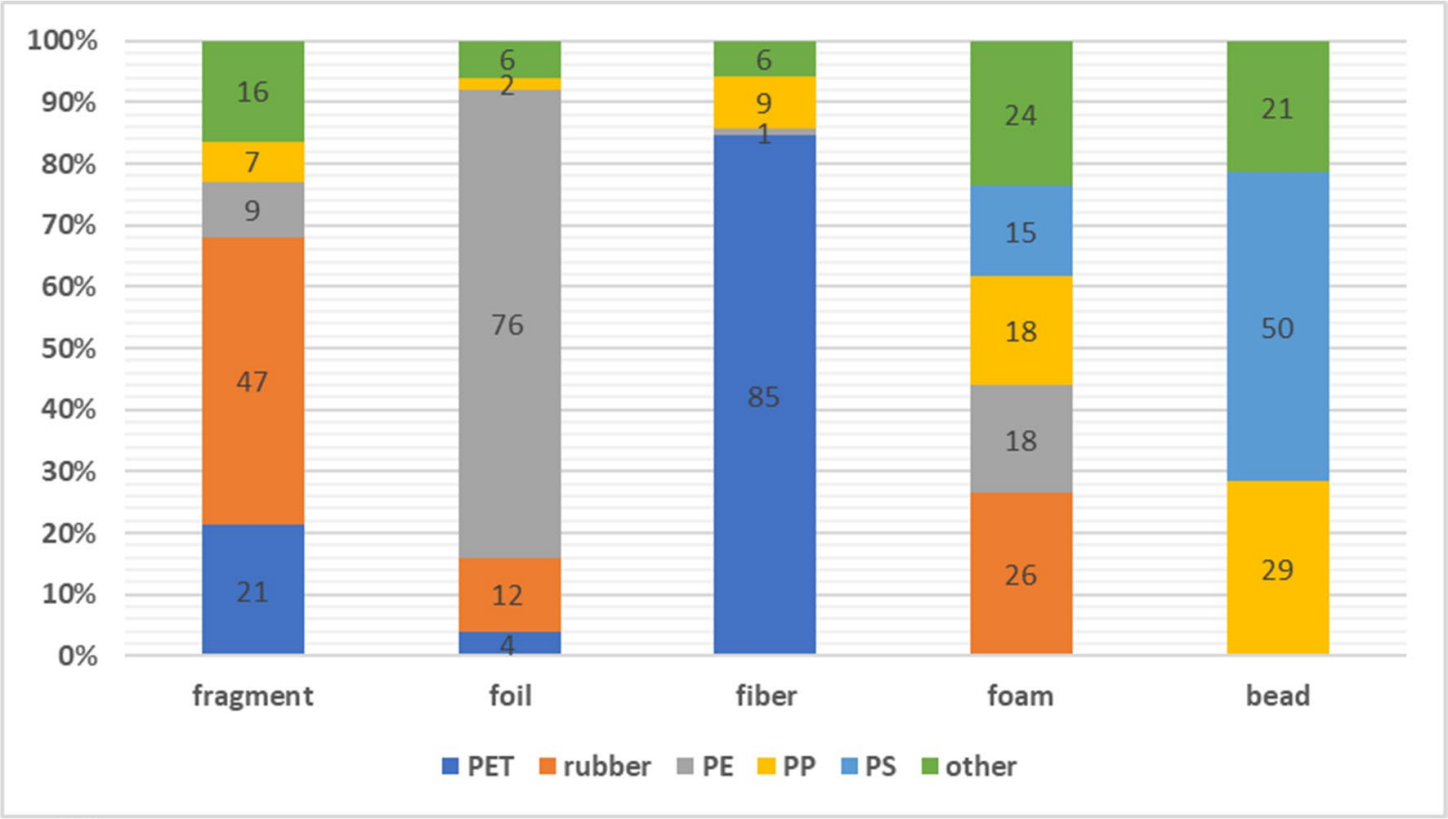
MP content in wastewater depending on its shape and polymer type

The largest number of MPs isolated from wastewater are fibres. Table 1 provides a numerical summary of all identified MPs in 100 L of the test sample. The large number of isolated fibres identified as PET influenced the highest content of this polymer in the sample. Washing synthetic textiles is considered one of the main sources of MPs in municipal WWTPs. Studies show that the number of fibres released into wastewater during washing ranges from 124 to 308 mg/kg of washed fabric, corresponding to 640,000 to 1,500,000 microfibres (De Falco et al. 2019). The small size of microfibre renders them susceptible to penetration of traditional filtration systems in WWTPs. Moreover, the efficacy of fibre removal by WWTPs is contingent upon the technology employed. In many cases, the efficiency is insufficient, contributing to the significant contamination of surface and marine waters (Michielssen et al. 2016). Analysis of the type of polymer-forming MPs, in relation to its shape, showed that 85% of all fibres identified were PET (Fig. 4). The remaining fibres were PP (9%), PCT (3%), polyamide (2%), and PE and polyacrylate (1% each). For example, a study by Šaravanja et al. indicates that MP fibres from PET and polyamides are present in significant quantities in wastewater, mainly due to washing processes for synthetic clothing (Šaravanja et al. 2022). The frequency of detection of PET shows its durability and ability to survive in wastewater treatment processes, which clearly confirms the fact that it originates from synthetic fabrics that people use every day (Militký et al. 2023).

**Table 1:** MP amount depending on the shape and type of polymer

	*Shape*	*Fragment*	*Foil*	*Fibre*	*Foam*	*Bead*	*Total*
*Type of polymer*	PET	29	2	149	-	-	180
	Rubber	63	6	-	9	-	78
	PE	12	38	2	6	-	58
	PP	9	1	15	6	4	35
	PS	-	-	-	5	7	12
	Silicon	5	2	-	3	2	12
	PUR	7	1	-	3	-	11
	PCT	-	-	6	1	-	7
	Polyamide (nylon)	2	-	3	-	-	5
	PE/PP	3	-	-	1	-	4
	Polyacrylate (PAM, PAN)	1	-	1	-	1	3
	EP	2	-	-	-	-	2
	PVC	2	-	-	-	-	2
	Total	135	50	176	34	14	409

Of the 13 polymers identified, as many as 11 are in the form of fragments. The variation in the composition of the fragments is most likely influenced by the fact that they are formed from the breakdown of larger plastics. The high content of MP fragments in wastewater is also influenced by the widespread use of plastics on a daily basis. Due to relatively low production costs and a wide range of properties, many everyday items are made of plastics (Hird 2022). The largest number of fragments (47%) is rubber pieces. The presence of rubber fragments in raw wastewater can be attributed to a number of sources. One significant contributor is the wear and tear of rubber tyres, which results in the production of rubber particles that are washed off roads and into sewer systems, particularly during rainstorms (Sommer et al. 2018). Tyres consist of different types of rubber, carbon black, fillers, metals, textiles, and other additives. Rubber accounts for approximately 40–60% of the total tyre composition (Evans and Evans 2006). Another source is the disintegration of rubber goods such as shoes, industrial belts, and household items that enter the sewage system through household drains (Abraham et al. 2011). The second most abundant polymer among the fragments is PET. PET is a common material used in the manufacture of beverage bottles and food packaging. PET fragments can originate from the waste of these materials, which are ultimately discharged into wastewater through sewage systems. Additionally, PET is used in a variety of industrial products, including electronic components and housings. These fragments can originate from manufacturing processes or industrial waste (Moog et al. 2019; Ngo et al. 2019).

Seventy-six percent of the foil were classified as PE. This is most likely due to the fact that PE is widely used in the production of reusable bags, agricultural film, food packaging film, etc. In 2021, the demand for PE by European plastics processors was 16.8% for low-density polyethylene (LDPE) and 12.6% for high-density polyethylene (HDPE). These are significant figures compared to the total demand for plastics in 2021, where only PP (19.8%) had a higher result (Plastics – the Facts 2021, 2021). PE foils are commonly used for food packaging, both in shops and in homes (Dirim et al. 2004). Residues of these materials can be accidentally or deliberately flushed down the drain. Items such as shopping bags, plastic packaging, or plastic covers can be a source of PE foils in wastewater, especially when disposed of incorrectly. Furthermore, as larger items made of PE, such as tarpaulins, outdoor toys, and garden furniture, degrade over time, they can release smaller fragments of PE film into the environment, which can then enter wastewater systems. Rubber film (12% of all film particles) is used in the construction industry. It is used for waterproofing, insulating vertical and horizontal parts of buildings, or insulating artificial water bodies (Martínez-Martínez et al. 2022). They can enter sewerage systems mainly through construction waste run-off. During the construction or demolition process, small particles and fragments of these materials can be washed away by rain or cleaning processes and enter sewers.

A large number of identified polymers are also found in foams. During the identification of foam particles, as many as eight different polymers were found. Twenty-six percent of these were identified as rubber. Rubber foam is employed in a variety of applications, including thermal and acoustic insulation in construction, automotive, and industrial contexts. Its insulating properties render it an ideal material for the sealing of doors, windows, and ventilation systems. Furthermore, its cushioning ability renders it a valuable packaging material, protecting products from damage during transport (Rostami-Tapeh-Esmaeil et al. 2021). PE and PP each accounted for 18% and PS 15%. PE and PP foams are commonly used for the safe packaging of electronic products, delicate components, as well as in the transportation of goods requiring protection from shock and damage (Crawford and Quinn 2017). They are also used in the manufacture of mattresses and pillows, where they provide comfort and support thanks to their ability to adapt to the shape of the body. The popularity of the use of these materials may result in an increase in the content of these particles in wastewater. PS is employed in a multitude of applications, most notably in the form of foam, as a packaging material, and in the manufacture of insulation boxes. Its lightweight, insulating properties, and low production cost render it a popular choice for the protection of products during transport and in food containers (Crawford and Quinn 2017). However, these same characteristics also make PS durable and resistant to biodegradation, which has led to its long-term presence in the environment. The daily use of PS in foamed food and industrial packaging contributes to an increase in the amount of this material in wastewater that enters sewer systems through domestic and industrial waste.

Beads (pieces with a spherical structure) accounted for just over 3% of the particles found in the wastewater. Fifty percent of these were identified as PS beads. It is likely that the degradation of foam composed of many pre-developed PS beads leads to the presence of this polymer in the environment in the form of small beads. PS in bead form is a common filler material used in packaging, particularly in courier shipments and the transportation of fragile items (Crawford and Quinn 2017). It is possible that residual materials may be washed or flushed down the drain when packaging is discarded, either accidentally or unknowingly. In industries where PS is processed or recycled, fine beads and dust can be emitted into the environment. Such particles can then be washed down the drain with rainwater. Furthermore, PS beads are lightweight and can be readily dispersed by wind. Such materials can enter the sewer system through drains, particularly in urban areas. Granules produced from plastics such as PE, PP, PS, or PET are used as ingredients in personal care products, including body washes, facial scrubs, or in cleaning products (O'Connor et al. 2019). They find their way directly into wastewater treatment systems. Despite the existence of regulations aimed at reducing the use of plastics in cosmetic products, such as bans on microbeads, they are still found in the ingredients of some products. These regulations, although aimed at reducing the environmental impact of plastics, are not always effective due to differences in implementation and enforcement at national and international levels.

**Fig. 5:**
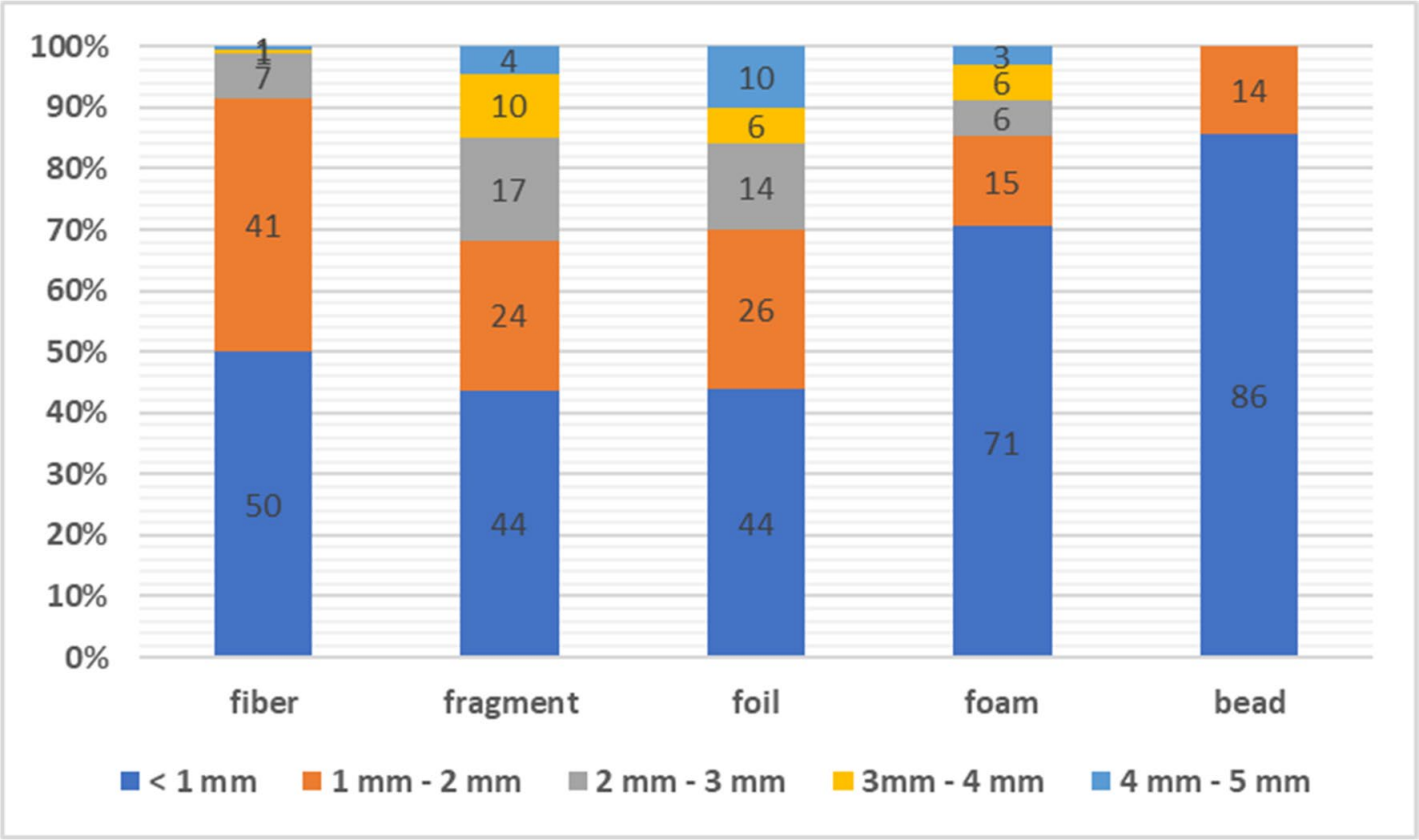
MP content in wastewater depending on its shape and size

Figure 5 shows the quantities of MPs according to their shape and size. The presence of fibres was confirmed in each fraction. Fifty percent of all fibres were within the size range of less than 1 mm. A further 41% fell within the size range of 1 mm to 2 mm. The length of the synthetic fibres depends on the type of material and the purpose for which it is intended. The fibres analysed are in the smallest sizes identified which may suggest fragmentation of longer fibres in the textile washing process (Periyasamy and Tehrani-Bagha 2022). The number of fragments in all size fractions is comparable. The variation in both the type of polymers detected and the size of the fragments may be due to the large number of plastic products used. Everyday objects under mechanical stress lose their integrity, resulting in fragmentation. In the form of small fragments (MP), they end up directly via the sewage system in the WWTPs (Song et al. 2024). In the case of films, foams, and beads, it has been observed that their content increases as the size of the fragments decreases. Foils are usually produced in large sizes. Their strength varies depending on the material used in their production. Sometimes the strength of the foil is not properly matched to the weight, causing the foil to break into smaller pieces. More than half of the foams (71%) in the effluent were classified as fractions in the range smaller than 1 mm. The same is true for beads. Eighty six percent of all beads fall within the size range smaller than 1 mm. Bead-shaped microparticles are deliberately produced in microscopic sizes. They are added to commercial products, such as face wash or toothpaste, and their presence has been confirmed in the laboratory (Duis and Coors 2017; Dąbrowska et al. 2022).

## Conclusion

The content of MPs in raw wastewater is the result of many factors, which can be divided into several main categories:

1. Consumption of plastic products—the high consumption of plastic items, including disposable packaging, cosmetic products with microbeads, and synthetic textiles (such as clothing), directly translates into the amount of MPs going into wastewater.
2. Industrial and manufacturing processes—industries that use plastics, including manufacturing, processing, and recycling, can generate waste containing MPs, which are then flushed down the drain.
3. Inadequate waste management—lack of proper recycling systems and inadequate waste handling can lead to plastic waste ending up in sewer systems instead of proper treatment facilities.
4. Erosion of plastic products—many items made of plastic, such as garden furniture, toys, and tools, degrade when exposed to the elements. These fragments can be washed into drains when it rains.
5. Washing synthetic clothing—washing synthetic fabrics, such as polyester or nylon, releases plastic microfibres that are small enough to easily pass through the filtration systems of WWTPs and enter the aquatic environment.

The effluent from the studied WWTP is very diverse in its physical and chemical characteristics of the MPs isolated. Analysis of potential sources indicates that the largest source of MPs in municipal WWTP may be the washing of synthetic fabrics. WWTPs are exposed to an influx of MPs of varying composition and size, which will further complicate the development of effective methods to treat wastewater from MPs. However, by understanding the sources of MPs, wastewater treatment technologies can be adapted to more effectively remove these specific particles. By understanding where MPs come from, strategies can be developed to prevent them from entering wastewater in the first place. These may include better industrial practices, changes in product design, and informed consumer choices. Accurate knowledge of the sources of MPs can support the development and implementation of regulations to reduce their emissions. This in turn can contribute to greater social and corporate environmental responsibility. In addition, understanding the sources of MPs in wastewater will help to raise public awareness of their environmental impact and the importance of sustainable waste and resource management.

The study covers one of Poland’s municipal WWTP, but the levels of MPs in other plants may be similar. It is therefore necessary to identify the sources of MPs for other municipal WWTPs. In addition, knowledge of the sources of MPs entering municipal WWTPs will stimulate further research on their environmental and health impacts, and on methods of disposal and treatment.

## Data Availability

Not applicable.
